# Right Gonadal Vein Thrombosis in Acute Intermittent Porphyria: A Rare Thrombotic Complication

**DOI:** 10.7759/cureus.105498

**Published:** 2026-03-19

**Authors:** Jyothsna Goranti, Daniel Neri Rosario, Kelly Chohonis

**Affiliations:** 1 Internal Medicine, Texas Tech University Health Sciences Center El Paso, El Paso, USA

**Keywords:** acute intermittent porphyria, anticoagulation, differential diagnosis for abdominal pain, gonadal vein thrombosis, porphyria-safe antibiotics, pulmonary embolism (pe), septic pelvic thrombophlebitis, venous thromboembolism (vte)

## Abstract

Acute intermittent porphyria (AIP) is a rare metabolic disorder characterized by neurovisceral manifestations, most commonly severe abdominal pain. Thrombotic complications are not typically associated with AIP and are infrequently reported in atypical venous territories. Gonadal vein thrombosis (GVT) is an uncommon condition most frequently observed in the postpartum period or in association with pelvic infections and malignancy; its occurrence in patients with AIP is exceptionally rare. GVT is a potentially serious form of deep vein thrombosis, often underdiagnosed due to its nonspecific presentation. We report the case of a 53-year-old woman with genetically confirmed AIP, a history of recurrent venous thromboembolism, and poorly controlled type 2 diabetes mellitus, who presented with acute lower abdominal pain radiating to the groin and back, accompanied by gastrointestinal symptoms. Contrast-enhanced computed tomography demonstrated an acute right GVT and a concurrent nonocclusive pulmonary embolism. Initial laboratory investigations revealed hyponatremia, mild transaminitis, and hyperglycemia. The overlapping abdominal manifestations of AIP initially complicated the diagnostic process; however, the presence of focal groin and back pain prompted further evaluation, leading to a timely diagnosis. The patient was managed with porphyria-safe antibiotics, therapeutic anticoagulation, and supportive care, resulting in clinical improvement. This case underscores the importance of maintaining a high index of suspicion for uncommon thrombotic complications in patients with AIP who present with new or atypical pain patterns. Early recognition and careful selection of porphyria-safe therapeutic strategies are critical to prevent complications and optimize clinical outcomes.

## Introduction

Acute intermittent porphyria (AIP) is a rare autosomal dominant disorder classified among a group of inherited genetic disorders characterized by defective enzymes of the heme biosynthesis pathway, collectively referred to as porphyrias [1]. According to the European Porphyria Network (EPNET), the prevalence of AIP in Europe is estimated to be approximately 1 in 20,000 individuals [2,3]. In Euro-Western populations, the prevalence of a disease-causing mutation is estimated to be 1 in 2,000; however, the prevalence of clinically symptomatic disease is significantly lower, ranging from 0.5 to 10 per 100,000 individuals [1,4,5]. Women are more frequently affected than men, with a reported female-to-male ratio ranging from 1.5:1 to 2:1, and symptoms most commonly manifest between the ages of 20 and 40 years [1,5].

AIP is caused by a deficiency of porphobilinogen deaminase (PBGD), also known as hydroxymethylbilane synthase (HMBS), the third enzyme in the heme biosynthesis pathway. This enzyme defect leads to the accumulation of neurotoxic heme precursors such as aminolevulinic acid (ALA) and porphobilinogen (PBG) [1,5]. Despite its autosomal dominant inheritance, clinical penetrance is low, and approximately 80-90% of individuals carrying an *HMBS* mutation remain asymptomatic [4,5]. Acute attacks may be precipitated by various triggers, including infections, fasting, a low-carbohydrate diet, alcohol consumption, or hormonal fluctuations, and certain medications, all of which increase hepatic ALA synthase activity and subsequent precursor accumulation [5].

The clinical manifestations of AIP are heterogeneous, ranging from mild and infrequent episodes to severe, life-threatening attacks. Abdominal pain is the cardinal symptom, occurring in more than 80% of patients, and is frequently accompanied by nausea, vomiting, and constipation [1,3]. Additional manifestations may include hyponatremia, muscle weakness, peripheral neuropathy, neuropsychiatric disturbances, autonomic dysfunction, and, less commonly, fever [1,4]. Long-term complications include chronic kidney disease, hepatocellular carcinoma, persistent neuropathy, and chronic pain syndromes [4,5]. Although thrombotic complications are not typical features of AIP, they may occur secondary to factors such as central venous catheterization, immobility, infection, or underlying hypercoagulable states [6,7].

Gonadal vein thrombosis (GVT) is an uncommon condition most frequently reported in the postpartum period or in association with sepsis, pelvic infections, malignancy, and systemic hypercoagulable disorders [6,7]. While rare, GVT can lead to serious complications, including extension into the inferior vena cava or pulmonary embolism if not promptly recognized and treated [7]. Clinical presentation is often nonspecific and may mimic other causes of acute abdominal or pelvic pain, contributing to delayed diagnosis. Notably, approximately 80-90% of cases involve the right gonadal vein [7].

We present a rare case of acute right GVT in a patient with AIP and recurrent deep vein thromboses (DVTs), highlighting the diagnostic challenges posed by overlapping abdominal symptoms and the complexities of managing thromboembolic disease in the context of porphyria.

## Case presentation

A 53-year-old woman with a known history of AIP, recurrent DVTs, and poorly controlled type 2 diabetes mellitus presented with a one-week history of progressively worsening lower abdominal pain radiating to the right groin and back. The pain was sharp, constant, and gradually intensifying. Associated symptoms included nausea and diarrhea for three days, fever for two days, and vomiting for one day. The patient initially believed she was experiencing an AIP flare, as the abdominal discomfort resembled prior episodes. She also reported recurrent brown blistering skin lesions over the lower abdomen during previous attacks. During this admission, she noted an ulcerated lesion in the right lower abdominal region, where the pain was most pronounced.

On arrival, she was febrile but hemodynamically stable. Physical examination revealed a non-distended abdomen with diffuse tenderness to palpation, most severe in the right groin region. Dermatologic examination demonstrated multiple circular hyperpigmented macular lesions over the lower abdomen, as well as an ulcerated lesion in the right lower quadrant without purulent drainage or surrounding fluctuance. The detailed laboratory findings obtained on admission are summarized in Table 1.

**Table 1:** Laboratory findings on admission. ALT: alanine transaminase; AST: aspartate transaminase

Laboratory test	Patient value	Reference range
White blood cell count	8.7 × 10⁹/L	4.0–11.0 × 10⁹/L
Hemoglobin	16.2 g/dL	12.0–15.5 g/dL (female)
Platelet count	271 × 10⁹/L	150–400 × 10⁹/L
Sodium	129 mEq/L	135–145 mEq/L
Corrected sodium	132 mEq/L	135–145 mEq/L
Potassium	3.6 mEq/L	3.5–5.0 mEq/L
Creatinine	0.7 mg/dL	0.6–1.1 mg/dL (female)
Glucose	348 mg/dL	70–100 mg/dL (fasting)
HbA1c	12.1%	<5.7% (normal)
AST	44 U/L	10–40 U/L
ALT	38 U/L	7–35 U/L
Alkaline phosphatase	128 U/L	44–147 U/L
Lactate	1.2 mmol/L	0.5–2.2 mmol/L
Urinalysis – glucose	4+	Negative
Urinalysis – urobilinogen	Normal	0.2–1.0 EU/dL

**Figure 1:**
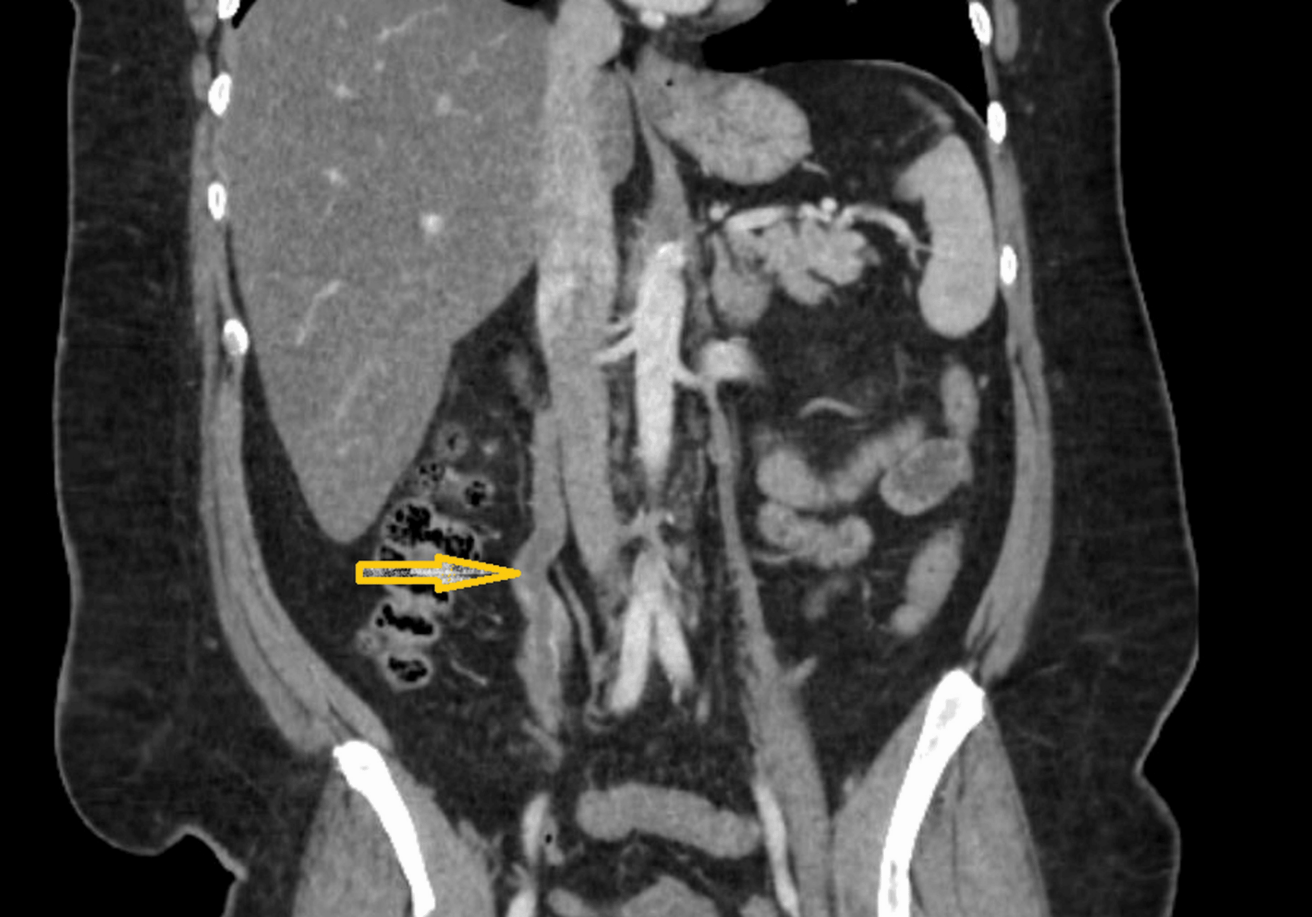
Contrast-enhanced computed tomography (CT) of the abdomen showing acute right gonadal vein thrombosis. Coronal contrast-enhanced CT image demonstrating a filling defect within the right gonadal vein (yellow arrow), consistent with acute thrombosis.

**Figure 2:**
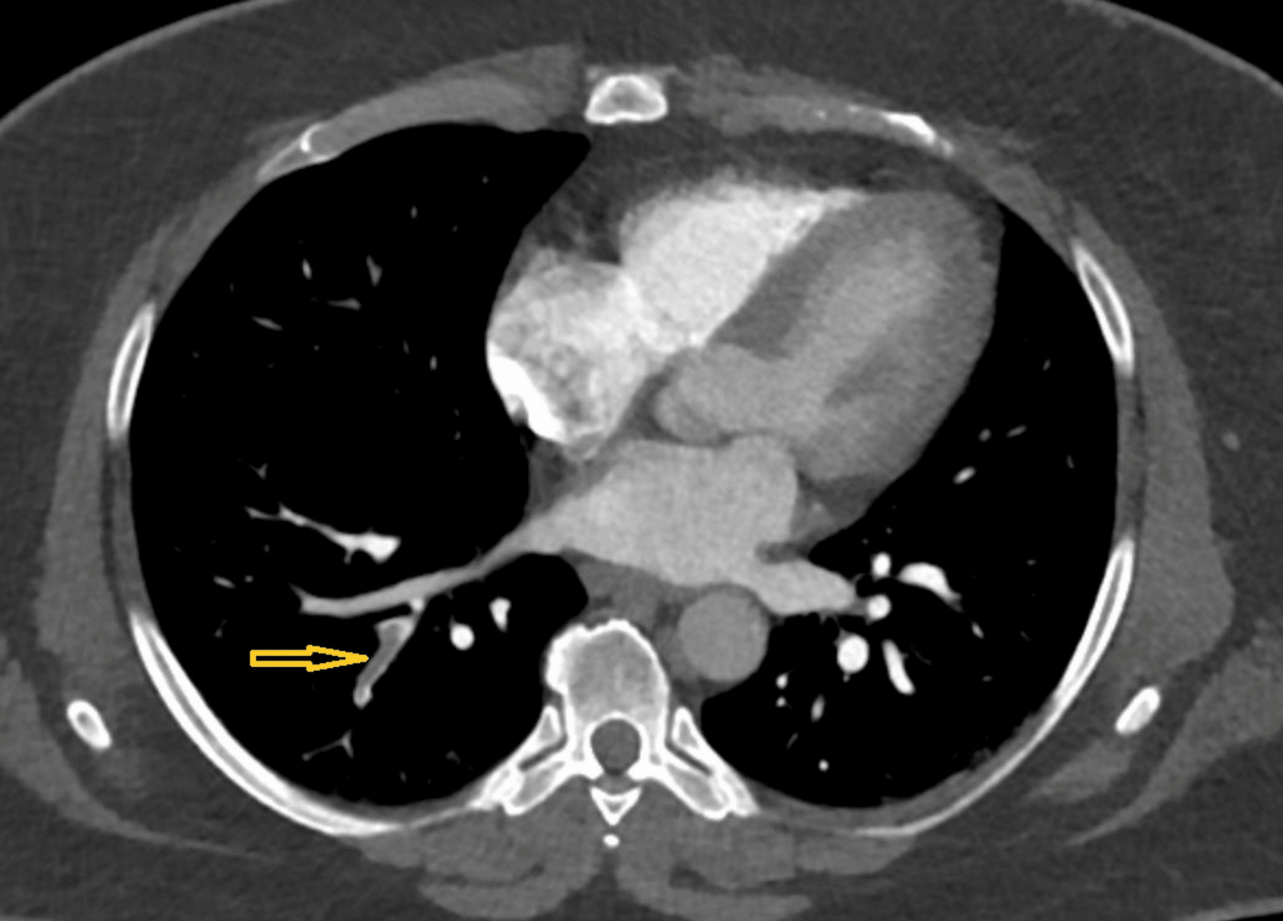
Contrast-enhanced computed tomography (CT) pulmonary angiography demonstrating acute pulmonary embolism. Axial CT pulmonary angiography image showing a nonocclusive filling defect within a segmental branch of the right lower lobe pulmonary artery (arrow), consistent with acute pulmonary embolism. No evidence of right heart strain is observed.

Contrast-enhanced computed tomography (CT) of the abdomen and pelvis revealed an acute right GVT (Figure 1), significant attenuation of the external iliac veins, and extensive venous collaterals within the abdominal wall and inguinal region. CT angiography (CTA) of the chest demonstrated an acute, nonocclusive pulmonary embolism involving segmental branches of the right lower lobe, with a right ventricular/left ventricular ratio <1 and no evidence of right heart strain (Figure 2).

Given the presence of fever and examination findings, septic pelvic thrombophlebitis was initially suspected, and obstetrics/gynecology was consulted. Therapeutic anticoagulation with enoxaparin 90 mg twice daily was initiated.

Due to persistent fever and tenderness over the abdominal ulcer, empiric antibiotic therapy with vancomycin and ampicillin-sulbactam was initiated; both agents were selected based on their safety profile in AIP. However, blood cultures remained negative, no clear infectious source was identified, and antibiotics were subsequently discontinued once an infectious etiology was excluded. Pain management was challenging due to her porphyria; however, she was safely treated with intravenous hydromorphone and acetaminophen. Her poorly controlled diabetes mellitus was addressed with initiation of basal insulin (glargine) and sliding-scale insulin therapy.

During hospitalization, her abdominal and back pain gradually improved with anticoagulation and supportive care. Fever resolved, and blood cultures remained negative. Given her history of recurrent thrombosis and evidence of chronic venous remodeling, long-term anticoagulation was indicated. She was transitioned to warfarin 5 mg daily (considered safe in prophyria), with a short five to seven-day apixaban bridge to maintain therapeutic anticoagulation while awaiting international normalized ratio stabilization. She was discharged in stable condition with close outpatient follow-up arranged with hematology, gynecology, and a porphyria specialty clinic.

## Discussion

This case illustrates a rare presentation of right GVT in a patient with AIP. Although this specific manifestation is uncommon, other thrombotic complications have been described in porphyria, including inferior vena cava (IVC) thrombosis [8]. Because AIP is primarily recognized for its neurovisceral manifestations, the potential association with venous thromboembolism (VTE) may be underappreciated, contributing to delayed recognition and misdiagnosis [5,7]. Emerging evidence suggests that patients with AIP may have an increased thrombotic risk, possibly related to metabolic and prothrombotic mechanisms such as hyperhomocysteinemia [9]. In the present case, the presence of focal groin and back pain, distinct from her prior AIP episodes, prompted further evaluation and ultimately led to the correct diagnosis.

GVT is most commonly associated with the postpartum state, pelvic malignancies, or systemic hypercoagulable conditions [7,8]. In this patient, a history of recurrent DVTs likely contributed to an underlying prothrombotic milieu, predisposing her to this atypical thrombotic event. A comprehensive thrombophilia workup was not performed in this case, which limits the ability to fully exclude underlying inherited or acquired hypercoagulable conditions contributing to her recurrent thrombotic events. However, her prior thrombotic history and acute illness likely contributed to a prothrombotic state. A retrospective cohort study by Alsharif et al. [6] in non-porphyria patients demonstrated that GVT may be complicated by pulmonary embolism and thrombus propagation into the IVC, both of which underscore the clinical significance of early diagnosis. Contrast-enhanced CT remains the diagnostic modality of choice due to its high sensitivity and ability to evaluate for concurrent complications. Alternative imaging modalities, including magnetic resonance venography and duplex ultrasonography, may be considered when contrast administration is contraindicated [10].

The nonspecific presentation of AIP frequently leads to diagnostic delays [11]. Measurement of urinary PBG and ALA during symptomatic periods remains the cornerstone of diagnosing acute attacks [5,12]. Genetic testing for mutations in the *HMBS* gene provides definitive confirmation and facilitates screening of at-risk family members [4]. Early identification of asymptomatic carriers enables counseling on trigger avoidance and preventive strategies [5].

Management of AIP requires prompt recognition, avoidance of precipitating factors, and careful medication selection [13]. Many commonly used antibiotics and analgesics are porphyrinogenic and may precipitate severe attacks [14]. In this case, vancomycin and ampicillin-sulbactam were selected due to their favorable safety profile in AIP; however, antibiotics were discontinued once an infectious etiology was excluded. Intravenous hemin remains the treatment of choice for acute attacks, as it suppresses hepatic ALA synthase activity and reduces the accumulation of neurotoxic intermediates [1]. Intravenous glucose may serve as adjunctive therapy in mild attacks [13]. For patients with recurrent attacks, givosiran, an RNA interference therapy targeting hepatic ALA synthase 1, has demonstrated efficacy in reducing attack frequency [15,16].

Management considerations in this patient extended beyond control of AIP. The presence of recurrent thrombosis and an atypical thrombotic site warranted long-term anticoagulation. A comprehensive VTE risk assessment is essential in patients with AIP who develop thrombotic events, particularly when additional risk factors are present. Given the limited data regarding thrombotic risk in AIP, further research is needed to clarify the mechanisms underlying hypercoagulability in this population and to identify biomarkers that may guide risk stratification and prophylactic strategies.

## Conclusions

This case underscores the importance of maintaining a high index of suspicion for GVT in patients with AIP who present with new, unexplained lower abdominal or groin pain. The overlap between typical AIP symptoms and manifestations of VTE can obscure diagnosis, potentially delaying appropriate management. Given the complexity of treating AIP in the setting of concurrent thromboembolic disease, a multidisciplinary approach is essential. Recognition of atypical thrombotic sites and careful selection of porphyria-safe therapeutic agents are critical for timely intervention and optimal clinical outcomes.
